# High damage tolerance of electrochemically lithiated silicon

**DOI:** 10.1038/ncomms9417

**Published:** 2015-09-24

**Authors:** Xueju Wang, Feifei Fan, Jiangwei Wang, Haoran Wang, Siyu Tao, Avery Yang, Yang Liu, Huck Beng Chew, Scott X. Mao, Ting Zhu, Shuman Xia

**Affiliations:** 1 Woodruff School of Mechanical Engineering, Georgia Institute of Technology, Atlanta, Georgia 30332, USA; 2 Department of Mechanical Engineering and Materials Science, University of Pittsburgh, Pittsburgh, Pennsylvania 15261, USA; 3 Department of Aerospace Engineering, University of Illinois at Urbana-Champaign, Urbana, Illinois 61801, USA; 4 Center for Integrated Nanotechnologies, Sandia National Laboratories, Albuquerque, New Mexico 87185, USA

## Abstract

Mechanical degradation and resultant capacity fade in high-capacity electrode materials critically hinder their use in high-performance rechargeable batteries. Despite tremendous efforts devoted to the study of the electro–chemo–mechanical behaviours of high-capacity electrode materials, their fracture properties and mechanisms remain largely unknown. Here we report a nanomechanical study on the damage tolerance of electrochemically lithiated silicon. Our *in situ* transmission electron microscopy experiments reveal a striking contrast of brittle fracture in pristine silicon versus ductile tensile deformation in fully lithiated silicon. Quantitative fracture toughness measurements by nanoindentation show a rapid brittle-to-ductile transition of fracture as the lithium-to-silicon molar ratio is increased to above 1.5. Molecular dynamics simulations elucidate the mechanistic underpinnings of the brittle-to-ductile transition governed by atomic bonding and lithiation-induced toughening. Our results reveal the high damage tolerance in amorphous lithium-rich silicon alloys and have important implications for the development of durable rechargeable batteries.

## Abstract (web summary)

Mechanical degradation is an undesired behaviour for battery electrode materials such as lithiated silicon. Here, the authors perform *in situ* nanomechanical experiments and atomistic modelling to reveal the damage tolerance of electrochemically lithiated silicon.

## Graphical abstract

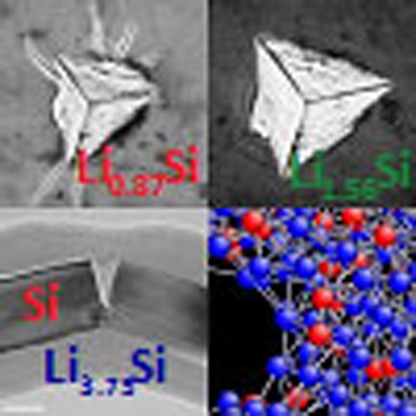

Energy storage with high performance and low cost is critical for applications in consumer electronics, zero-emission electric vehicles and stationary power management^1^,^2^. Lithium-ion batteries (LIBs) are the widely used energy storage systems due to their superior performance^3^,^4^,^5^. The operation of a LIB involves repeated insertion and extraction of lithium (Li) ions in active battery electrodes, which are often accompanied with considerable volume changes and stress generation^6^,^7^,^8^,^9^,^10^,^11^,^12^. In the development of next-generation LIBs, mechanical degradation in high-capacity electrode materials arises as a bottleneck. Such high-capacity electrode materials usually experience large volume changes (for example, up to about 280% for silicon (Si)), leading to high mechanical stresses and fracture of electrodes during electrochemical cycling^13^,^14^,^15^,^16^,^17^,^18^,^19^,^20^,^21^,^22^. Fracture causes the loss of active materials and yields more surface areas for solid electrolyte interphase (SEI) growth, both of which contribute to the fast capacity fade of LIBs.

To mitigate the mechanical degradation in LIBs, it is essential to quantitatively understand the electrochemically-induced mechanical responses and fundamental mechanical properties of high-capacity electrode materials. The past decade has witnessed a marked increase in studies on the mechanical behaviours of high-capacity electrode materials^6^,^7^,^8^,^9^,^10^,^11^,^12^,^13^,^14^,^15^,^16^,^17^,^18^,^19^,^20^,^21^,^22^. Focusing on the promise of Si as the next-generation anode material, significant progress has been made in the experimental measurement and modelling of lithiation/delithiation-induced stresses^11^,^23^, Li concentration-dependent modulus and hardness^24^,^25^,^26^, time-dependent creep^26^,^27^ and strain-rate sensitivities^28^. However, there is still a critical lack of fundamental understanding on the fracture-related properties^29^,^30^, which ultimately dictate the resistance of the electrode material to mechanical degradation and failure.

Here we report an integrated experimental and computational investigation on the damage tolerance of lithiated Si. We performed *in situ* nanomechanical bending test on a partially lithiated Si nanowire inside a transmission electron microscope (TEM). The result reveals a direct contrast between the low fracture resistance in the brittle core of pristine Si and the high damage tolerance in the ductile shell of fully lithiated Si. We conducted systematic measurements of the fracture toughness of lithiated Si thin films with nanoindentation. The results show a rapid brittle-to-ductile transition of fracture in Li_*x*_Si alloys with increasing Li concentration. We also performed continuum finite element (FE) and molecular dynamics (MD) simulations to interpret these experimental findings, thereby gaining mechanistic insights into the fracture mechanisms of lithiated Si. These results have important implications for the development of durable Si-based anodes for next-generation LIBs.

## Results

### *In situ* electrochemical and bending tests of Si nanowires

**Figure 1:**
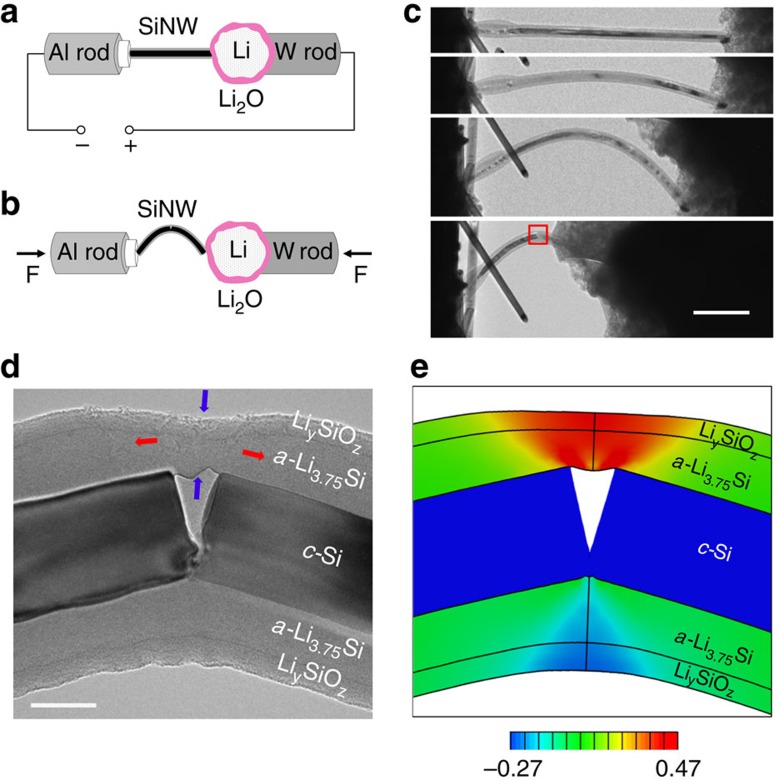
*In situ* electrochemical and bending test of a Si nanowire. (**a**) Schematic of the *in situ* TEM setup for a nano-sized electrochemical cell. (**b**) Schematic of the buckling and bending test of a partially lithiated Si nanowire subjected to the compressive force F. (**c**) Sequential TEM images showing the process of axial compression; bucking and bending of the partially lithiated Si nanowire gave rise to a sharply kinked region indicated by the red box (scale bar, 1 μm). (**d**) Zoom-in TEM image (that is, the red box region in **c**) showing the brittle fracture of the unlithiated *c*-Si core, as well as the large tensile deformation (red arrows) and lateral thinning (blue arrows) of the lithiated *a*-Li_3.75_Si shell (scale bar, 50 nm). (**e**) Finite element result showing the simulated elastic-plastic deformation in the nanowire that agrees with the TEM image in **d**. Colour contour reveals the distribution of axial strain in the lithiated *a*-Li_3.75_Si shell, with an extraordinarily large tensile strain of about 47% occurring in the free-standing part of *a*-Li_3.75_Si.

Figure 1a,b show the schematic illustrations of *in situ* electrochemical lithiation and fracture testing of a single Si nanowire inside a TEM. The nano-sized electrochemical cell^31^ consisted of a Si nanowire on Si substrate as the working electrode and a Li probe as the counter electrode; the native Li_2_O layer on Li surface served as a solid electrolyte. The <111>-oriented single crystal Si nanowire had an initial diameter of about 142 nm. To start the lithiation, the Li probe was brought into contact with the free end of the Si nanowire and then a bias voltage of −2 V was applied between the working and the counter electrodes. Because Li diffusion/reaction on the surface of Si is much faster than that in the bulk^14^, Li ions first migrated preferably along the free surface of the Si nanowire and then diffused towards its centre. In the bulk of the Si nanowire, lithiation proceeded in a core-shell mode, yielding a core of crystalline Si (*c*-Si) and a shell of amorphous Li_*x*_Si (*a*-Li_*x*_Si, *x*≈3.75). The core-shell interface was atomically sharp and oriented parallel to the longitudinal axis of the nanowire^12^. Across this interface, an abrupt change of Li concentration occurred, resulting in high compressive stresses near the core-shell interface (that is, behind the lithiation front)^32^. The lithiation-induced compression slowed down and eventually stalled the movement of the interface. At the end of the lithiation process, the diameter of the unlithiated *c*-Si core was 85 nm; the thicknesses of the *a*-Li_3.75_Si shell and the layer of lithiated surface oxide (Li_*y*_SiO_*z*_) were 47 and 17 nm, respectively.

After the lithiation experiment, the partially lithiated Si nanowire was subjected to *in situ* compression by the Li probe, as shown in Fig. 1c and Supplementary Movie 1. As the compressive load was increased, the nanowire buckled and subsequently bent, resulting in a single sharp kink in the nanowire. Figure 1d shows a zoom-in TEM image near the kinked region. It is seen that brittle fracture occurred in the unlithiated *c*-Si core, evidenced by the nearly flat fracture surfaces. The fracture strength of the *c*-Si core was determined to be about 7.7 GPa, which is consistent with the literature data on the strength of pristine <111>-oriented Si nanowires with similar sizes (see Supplementary Fig. 1 and related text). In contrast, the *a*-Li_3.75_Si shell underwent considerably large tensile deformation accompanied by pronounced lateral thinning, without observable damages such as cracking or shear banding. The lithiated surface oxide layer (Li_*y*_SiO_*z*_) remained coherent to the *a*-Li_3.75_Si shell and thus underwent similar straining as the latter. Figure 1e shows the result of a continuum FE simulation of large local deformation near the kinked region (see the modelling details in Supplementary Fig. 4 and Supplementary Methods). In the FE simulation, we assumed that the brittle fracture of the *c*-Si core was a much faster process than the elastic–plastic deformation in the *a*-Li_3.75_Si shell, such that the latter occurred after the crack in the *c*-Si core had been fully formed. As a result, the large tensile deformation in the *a*-Li_3.75_Si shell was primarily accommodated by the sliding at the core-shell interface, yielding a large crack opening in the *c*-Si core near the core-shell interface. The deformation morphology from the FE simulation (Fig. 1e) was in close agreement with that in the TEM image (Fig. 1d). The most striking observation from Fig. 1d,e is the large tensile (plastic) strain of ∼47% occurring in the *a*-Li_3.75_Si shell, with a concurrent large lateral contraction (that is, thinning) of ∼45%. Therefore, the *in situ* TEM experiment and FE simulation demonstrate that the amorphous Li-rich Si alloy (*a*-Li_3.75_Si) exhibits extraordinary damage tolerance, while *c*-Si is brittle as expected.

### Fracture toughness measurements of lithiated Si

**Figure 2:**
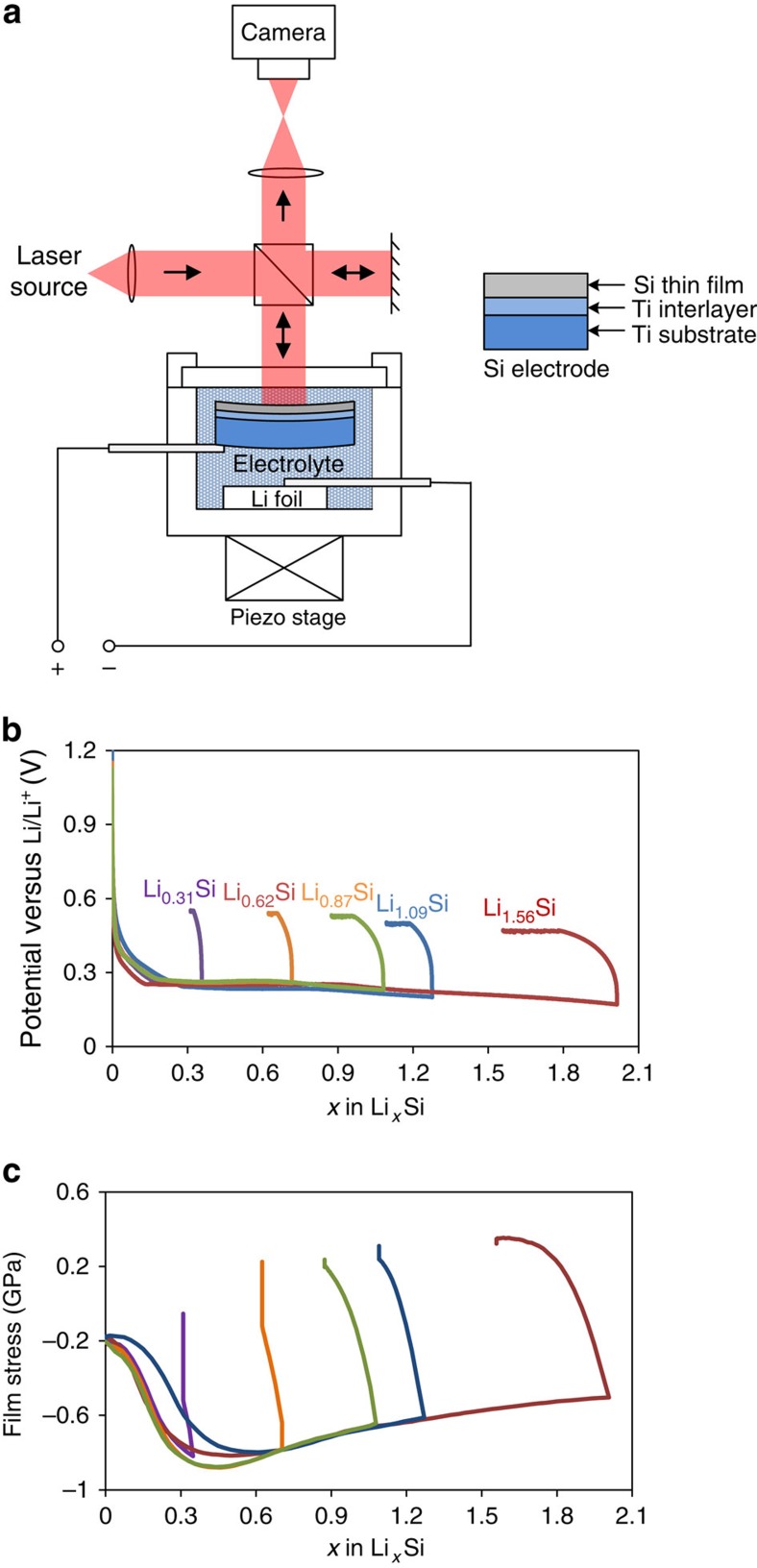
Electro–chemo–mechanical characterization of Si electrodes. (**a**) Schematic illustration of an electrochemical cell, consisting of a Si thin-film working electrode, a liquid electrolyte, and a Li foil counter electrode, as well as a Michelson interferometer setup for *in situ* film stress measurement. (**b**) Electrochemical profiles of five 325 nm Si electrodes that were galvanostatically lithiated and delithiated to various Li concentrations, followed by potentiostatic delithiation. (**c**) Evolution of the film stress in the five Si electrodes corresponding to the electrochemical profiles in **b**.

To quantitatively study the damage tolerance of electrochemically lithiated Si at different Li concentrations, we employed an in-house developed nanoindentation system (Supplementary Fig. 2) to characterize the fracture toughness of *a*-Li_*x*_Si, a property that describes the ability of a material to resist crack propagation^33^. The Si electrodes for nanoindentation tests were fabricated in a thin-film form by sputter deposition of 325 nm thick amorphous Si (*a*-Si) films onto flat titanium (Ti) substrates. Each Si electrode was lithiated in a custom-fabricated Teflon electrochemical cell, as illustrated in Fig. 2a, with a Li foil as the reference electrode. During the electrochemical testing of each *a*-Si electrode, a Michelson interferometer was used to measure the curvature change of the substrate from which the lithiation-induced biaxial film stress was deduced using Stoney's equation^34^.

Figure 2b,c show the electrochemical profiles and corresponding film stresses measured for five *a*-Si thin-film electrodes. The Li concentrations in these electrodes were controlled by galvanostatical lithiation at 20 μΑ cm^−2^ to different cutoff potentials. The sloping voltage profiles below 0.5 V versus Li/Li^+^ suggest the formation of a single-phase *a*-Li_*x*_Si during lithiation. As shown in Fig. 2c, all thin-film electrodes exhibited an initial compressive stress of about 200 MPa that resulted from the sputtering process. The compressive stress in the thin-film electrodes increased to 500–800 MPa at the end of lithiation, due to the substrate constraint on lithiation-induced volume expansion. This large residual compressive stress may impede crack growth during nanoindentation testing and thus affect the measurement of fracture toughness. To reduce the influence of those residual stresses, we first lithiated the *a*-Si electrode to a targeted Li concentration and then delithiated it to 0.25–0.3 V above the lithiation cutoff potential. During the delithiation, a small fraction of Li was extracted from the electrodes, resulting in small volume shrinkages so as to reverse the film stress from compression to tension. The tensile stress in the thin film was small, but found to be beneficial for promoting the nanoindentation-induced cracking and enabling the evaluation of fracture toughness of *a*-Li_*x*_Si. The effects of SEI formation on electrode cracking were estimated to be negligibly small^35^ and therefore were not considered in the fracture toughness measurement.

**Figure 3:**
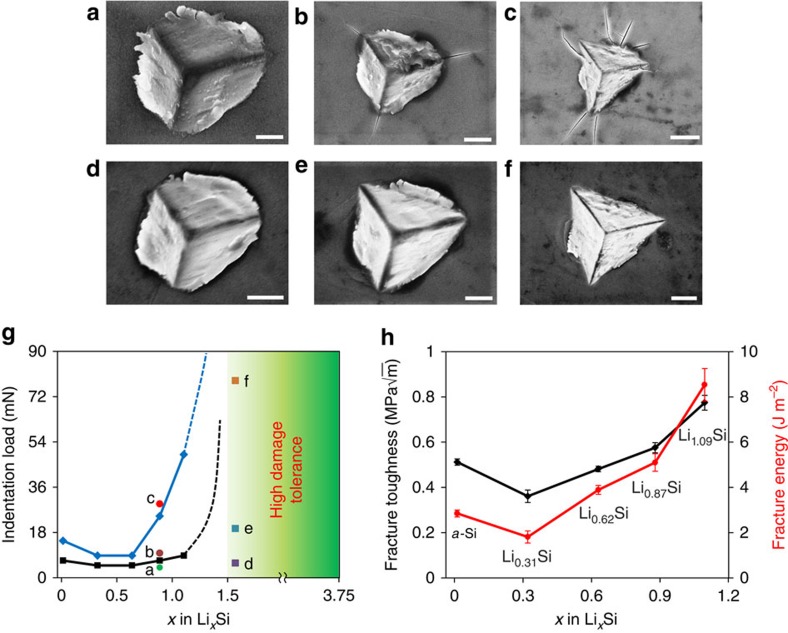
Fracture toughness measurement by nanoindentation. (**a**–**c**) SEM images of residual indents for a lithiated electrode of Li_0.87_Si, showing (**a**) no cracking, (**b**) radial cracking and (**c**) massive cracking subjected to different applied indentation loads (scale bar, 0.5 μm in **a**; 1 μm in **b**; 2 μm in **c**). (**d**–**f**) SEM images of residual indents for a lithiated Si electrode of Li_1.56_Si showing no cracking subjected to different applied indentation loads (scale bar, 1 μm in **d** and **e**; 2 μm in **f**). (**g**) The indentation loads (symbols) applied to the lithiated electrodes with different Li contents, corresponding to images in (**a**–**f**). The blue solid curve represents the upper load limit above which massive cracking occurred and the black solid curve the lower limit below which no crack was induced. The dashed lines show the qualitative trends interpolated from the data. (**h**) Fracture toughness and fracture energy of lithiated Si as a function of Li concentration.

To measure the facture toughness, the *a*-Li_*x*_Si thin-film electrodes were indented with a cube-corner indenter tip in an argon-filled glove box. After unloading of the indenter, residual indents and surrounding areas were imaged using a scanning electron microscope (SEM). Figure 3a–c show the nanoindentation results for a partially lithiated Si electrode with low Li concentration (Li_0.87_Si) under different indentation loads, which exhibit distinct cracking behaviours including (i) no cracking, (ii) indent corner cracking and (iii) massive cracking. Specifically, under a small load of 3.92 mN, residual plastic deformation at the permanent indent was observed without obvious cracking (Fig. 3a). As the load was increased to 9.8 mN, three radial cracks emanated from the sharp corners of the indent (Fig. 3b). A further increase in the indentation load to 29.4 mN resulted in massive cracking around the indent (Fig. 3c). Similar cracking behaviours were observed for low Li concentrations from *x*=0 to 1.09. However, as the Li:Si ratio was increased to above 1.56, no cracking was observed from indents for a wide range of indentation loads up to 93 mN, as shown in Fig. 3d–f. Critical indentation loads separating the above three regimes of cracking behaviours are plotted in Fig. 3g. The two critical load curves, respectively, represent the upper load limit above which massive cracking occurred and the lower limit below which no crack was induced. The upper load limit curve varies substantially with the Li concentration when *x* exceeds 0.6, indicating that the fracture toughness of lithiated Si starts to depend sensitively on the Li concentration. Similar drastic change of the lower load limit curve can be inferred as *x* varies from 1.09 to 1.56 (as indicated by the dashed line), since no cracking was observed for indentation loads up to 93 mN when *x*=1.56. Therefore, our results indicate that a brittle-to-ductile transition of fracture occurs when the Li composition falls in the range of *x*=1.09–1.56. For *x*≤1.09, the indentation loads in between the two limits induced well-developed radial cracks. We used these loads to evaluate the fracture toughness of *a*-Li_*x*_Si with the Morris nanoindentation model (see Supplementary Fig. 3 and Supplementary Methods for model details).

Figure 3h shows the measured fracture toughness, *ϰ*_Ic_, as a function of Li concentration. Also shown in this figure is the fracture energy, defined as *Γ*=*ϰ*_Ic_^2^/*E*, where *E* is Young's modulus of lithiated Si taken from the experimental measurement^25^. Figure 3h clearly reveals that the fracture toughness of *a*-Li_*x*_Si alloys depends sensitively on the Li concentration. For example, the fracture toughness and fracture energy of unlithiated *a*-Si are 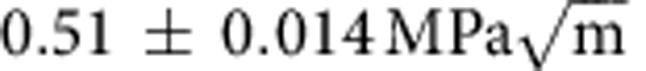 and 2.85±0.15 J m^−2^, respectively. These low values are typical of brittle materials with little fracture resistance^33^. As the Li concentration increases, the fracture resistance of lithiated Si first decreases slightly, indicating lithiation-induced embrittlement. This trend is in qualitative agreement with the recent *ab initio* calculations^36^ that show a small amount of Li insertion into Si substantially weakens Si–Si bonds, and hence reduces the surface energy of the material. However, upon further lithiation beyond *x*=0.31, both the measured fracture toughness and fracture energy increase substantially with increasing Li concentration, reaching 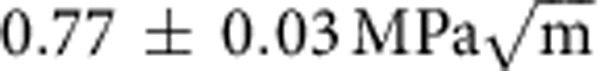 and 8.54±0.72 J m^−2^ for Li_1.09_Si, respectively. Since a further increase in Li concentration gave rise to large residual plastic deformation at and around indents without cracking (for example, Fig. 3d–f), we take the Li:Si ratio of *x*≈1.5 as the characteristic composition above which the brittle-to-ductile transition of fracture occurs in *a*-Li_*x*_Si alloys.

### Atomistic modelling of fracture in lithiated Si

**Figure 4:**
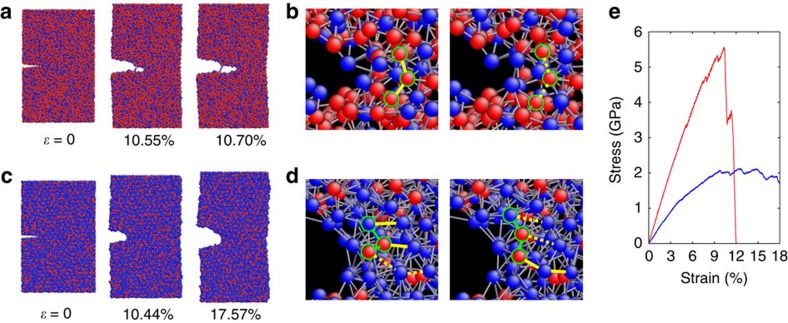
Molecular dynamics (MD) simulations. (**a**) MD snapshots of brittle fracture in Li-lean Si (*a*-Li_0.5_Si) at various stages of applied strain load *ɛ*, showing the growth of an atomically sharp crack. (**b**) Zoom-in images near the crack tip in strained *a*-Li_0.5_Si, showing the characteristic atomic processes of Si-Si bond breaking (from solid to dashed lines). (**c**) MD snapshots of ductile response in Li-rich Si (*a*-Li_2.5_Si) at various stages of applied strain load *ɛ*, showing the crack-tip blunting. (**d**) Zoom-in images near the crack tip in strained *a*-Li_2.5_Si, showing the characteristic atomic processes of bond breaking (from solid to dashed lines), formation (from dashed to solid lines) and rotation (angle change between two green lines). Si atoms are coloured by red and Li by blue in **a**–**d**. (**e**) Overall stress-strain responses of *a*-Li_0.5_Si (red curve) and *a*-Li_2.5_Si (blue curve).

To understand the experimentally measured brittle-to-ductile transition phenomenon, we performed MD simulations of deformation and fracture in *a*-Li_*x*_Si alloys using the reactive force field^23^ (see Methods section). In Fig. 4, we contrast the MD results by showing the brittle crack growth in Li-lean *a*-Li_0.5_Si versus the ductile crack blunting in Li-rich *a*-Li_2.5_Si. In both cases, MD simulations were performed for samples containing a sharp-edge pre-crack and subjected to a far-field tensile load. Figure 4a presents a sequence of MD snapshots showing brittle crack growth in *a*-Li_0.5_Si. In this case, the local tensile deformation near the crack tip is primarily accommodated by the stretching and breaking of Si–Si bonds, as shown in Fig. 4b. Due to the high fraction of strong covalent Si bonds, the bond alteration events are mostly discrete and disruptive, lacking bond reformation. A small damage zone forms near the crack tip, and it contains a high fraction of dangling bonds and atomic-sized voids. As the crack extends through such a damage zone, the crack faces become atomically rough, but the crack tip remains sharp, as seen from Fig. 4a. In contrast, Fig. 4c presents a sequence of MD snapshots showing the crack blunting in *a*-Li_2.5_Si. In this case, the local tensile deformation near the crack tip is accommodated by the stretching, rotation, breaking and frequent reformation of atomic bonds, as shown in Fig. 4d. Several Li atoms often collectively participate in a bond-switching process, so that the amorphous structure near the crack tip remains nearly homogenous without significant damage. As a result, the crack tip becomes considerably blunt without apparent crack growth, as seen from Fig. 4c. In addition, we compare the stress-strain curves during tensile straining of the above two pre-cracked samples in Fig. 4e; the fast brittle fracture in *a*-Li_0.5_Si is manifested as a sharp load drop, while the ductile crack blunting in *a*-Li_2.5_Si is characterized by a gradual load decrease, indicative of extensive plastic deformation near the crack tip. The above MD results of brittle versus ductile behaviours in Li-lean and Li-rich *a*-Li_*x*_Si alloys are consistent with the trend in nanoindentation measurements. Hence, our MD study reveals a plausible atomistic mechanism of brittle-to-ductile transition in *a*-Li_*x*_Si alloys, namely, the decreasing fraction of strong covalent Si bonding and the concomitant increasing fraction of delocalized metallic Li bonding give rise to an alteration of the dominant atomic-level processes of deformation and fracture with increasing Li concentration.

## Discussion

The above experimental and modelling results underscore the notion of the high damage tolerance of amorphous Li-rich Si alloys and thus have important implications for the design of durable Si-based anodes for next-generation LIBs. Recently, lithiation experiments have been conducted with *a*-Si nanoparticles with a wide range of diameters up to 870 nm^18^, as well as with *a*-Si pillars of a few microns in diameter^37^. In those experiments, no cracking and fracture were observed in lithiated *a*-Si electrodes. However, it has been shown that the surface layers of lithiated *a*-Si particles and wires should have undergone large hoop tensile deformation resulting from the two-phase lithiation and associated volume expansion at curved phase boundaries^17^,^18^. Given the large hoop tension and accordingly high driving force of fracture, the lack of observed cracking^37^,^38^ implies that the fully lithiated *a*-Si should be mechanically robust. In this work, our *in situ* TEM and nanoindentation experiments collectively provide direct evidence for the high damage tolerance (that is, high tensile ductility and high fracture toughness) of Li-rich Si alloys, which is the essential mechanistic information for the design of durable Si-based electrodes. In addition, the quantitative fracture characteristics obtained in our work constitute an important input for optimizing the microstructures and lithiation/delithiation windows of Si-based LIBs. Incidentally, the mechanical robustness of lithiated *a*-Si is in contrast with the commonly observed surface cracking and fracture after lithiation in large *c*-Si nanostructures^16^,^19^. The ease of fracture in lithiated *c*-Si has been attributed to a crystallographic effect on the lithiation anisotropy, resulting in inhomogeneous phases and deformation near the interface between anisotropically lithiated domains^39^. For *a*-Si electrodes, there is no such lithiation anisotropy. As a result, both the lithiated phases and deformation processes in *a*-Si particles and wires are more homogenous than those in *c*-Si counterparts, which contribute to the high mechanical robustness of the former.

To conclude, we have integrated experiments and modelling to reveal the high damage tolerance of electrochemically lithiated Si electrodes. The *in situ* TEM experiment on a partially lithiated Si nanowire showed a striking contrast of the brittle fracture in the unlithiated Si core versus the ductile tensile deformation in the lithiated Si shell. The nanoindentation testing of amorphous lithiated Si alloys indicated a drastic increase of fracture toughness as the Li to Si ratio was increased to above 1.5. Our atomistic simulations elucidated the mechanistic underpinnings of the brittle-to-ductile transition in terms of atomic bonding and lithiation-induced toughening. The quantitative characterization and mechanistic understanding of high damage tolerance of Li-rich Si alloys represent a critical step towards the rational design of durable Si-based electrodes for next-generation LIBs. Broadly, our integrated experimental and modelling approach can be applied to the mechanical characterization of a wide range of electrochemically active materials for energy storage applications.

## Methods

### Thin-film electrode fabrication and electrochemical cell assembly

Single-side polished titanium (Ti) plates with a thickness of 0.5 mm were used as substrates for fabrication of thin-film electrodes, and they also served as current collectors for electrochemical measurements. Before a Ti substrate was placed inside the deposition system (Denton Discovery RF/DC Sputterer), it was etched with 5% HCl to remove the surface Ti oxide. A 26 nm Ti thin film was first sputtered onto the polished side of the Ti substrate, followed by the deposition of a 325 nm thick Si film. The Ti interlayer, serving as an adhesion-promoting layer, was prepared by DC sputtering of a Ti target (3" diameter disc, 99.995% Ti, Kurt J. Lesker Co., Livermore, CA) at a power of 53 W and a pressure of 6.29 mTorr of argon. The Si film was prepared by RF magnetron sputtering of an Si target (3" diameter disc, 99.995% Si, Kurt J. Lesker Co., Livermore, CA, USA) at a power of 200 W and a pressure of 5 mTorr of argon. Previous studies have shown that Si thin films formed under these sputtering conditions are amorphous^40^.

After Si was deposited on the polished side of the Ti substrate, a thin layer of polydimethylsiloxane (PDMS) was coated on the unpolished side of the Ti substrate to prevent the formation of an SEI layer on the Ti surface. The Si film-coated Ti electrode was then assembled into a custom-fabricated Teflon electrochemical cell with a glass window (Fig. 2a) inside an argon-filled glove box that was maintained at <0.1 ppm of O_2_ and H_2_O. A Li foil was used as the reference/counter electrode, and 1 M of lithium hexafluoro-phosphate (LiPF_6_) in 1:1:1 (weight %) ratio of ethylene carbonate (EC) to dimethyl carbonate (DMC) to diethyl carbonate (DEC) was used as the electrolyte.

### Electrochemical and *in situ* film stress measurement

The electrochemical measurement was conducted with a battery tester (UBA 5, Vencon Technologies, Ontario, Canada). Five Si electrode samples were first lithiated galvanostatically at 20 μΑ cm^−2^ with pre-determined cutoff potentials. The lithiation was followed by delithation at the same current density until potentials of 0.2–0.3 V versus Li/Li^+^ above the cutoff potentials for lithiation were reached. Subsequently, delithiation was continued potentiostatically until the current was reduced below 0.2 μΑ cm^−2^. The Si electrodes were then removed from the cell inside the glove box and thoroughly cleaned by rinsing them with DMC. Stress evolution in the Si films during lithiation and delithiation was measured by monitoring the substrate curvature with a Michelson interferometer. The biaxial film stress was deduced from the measured curvature using Stoney's equation^34^.

### Nanoindentation experiments

The fracture toughness of lithiated Si electrodes was measured by an in-house developed nanoindentation system inside an argon-filled glove box (Supplementary Fig. 2). Peak loads, ranging from 1 to 93 mN at constant loading and unloading rates of 500 μN s^−1^, were used during nanoindentation tests. Ten indents at each load were made for each Si electrode sample and spaced 100 μm apart. The indented Si electrodes were imaged by a Zeiss Ultra60 FE-SEM (Carl Zeiss Microscopy, LLC, North America, Peabody, MA) operated at an accelerating voltage of 5 kV. During the sample transfer from the glove box to the SEM chamber, the lithiated Si electrodes were coated with a thin layer of anhydrous DMC to avoid the reaction of lithiated Si with ambient oxygen and moisture.

### Molecular dynamics simulations

Molecular dynamics simulations were performed using the large-scale atomic molecular massively parallel simulator (LAMMPS). Atomic interactions were modelled by a reactive force field developed for Li–Si alloys^23^. The *a*-Li_*x*_Si samples were prepared from melting-and-quenching simulations with a quench rate of 2 × 10^12^ K s^−1^. In these simulations, the initial structures were created by randomly inserting Li atoms into a crystalline Si lattice. After quenching at zero stresses, the *a*-Li_*x*_Si samples had the final dimensions of ∼16 × 10 × 1.5 nm. A sharp crack with a length of ∼3 nm was created in each sample. The pre-cracked samples were then loaded in tension with a strain rate of 5 × 10^8^ s^−1^, while the system temperature was maintained at 5 K. Periodic boundary conditions were applied along the loading and the thickness directions. A plane-strain condition was imposed by fixing the thickness of the simulation box. The time step in all the molecular dynamics simulations was 1 fs.

## Additional information

**How to cite this article:** Wang, X. *et al*. High damage tolerance of electrochemically lithiated silicon. *Nat. Commun.* 6:8417 doi: 10.1038/ncomms9417 (2015).

## Supplementary Material

Supplementary Information Supplementary Figures 1-4, Supplementary Methods and Supplementary References

Supplementary Movie 1 The buckling and bending test of a partially lithiated Si nanowire subjected to compressive loading.
