# Supplementary material for: High damage tolerance of electrochemically lithiated silicon
Source: Nat Commun. 2015 Sep 24;6:8417. doi: 10.1038/ncomms9417 (PMC4598720; doi:10.1038/ncomms9417)
Supplement: Supplementary Information — Supplementary Figures 1-4, Supplementary Methods and Supplementary References [file ncomms9417-s1.pdf]

Supplementary Figures

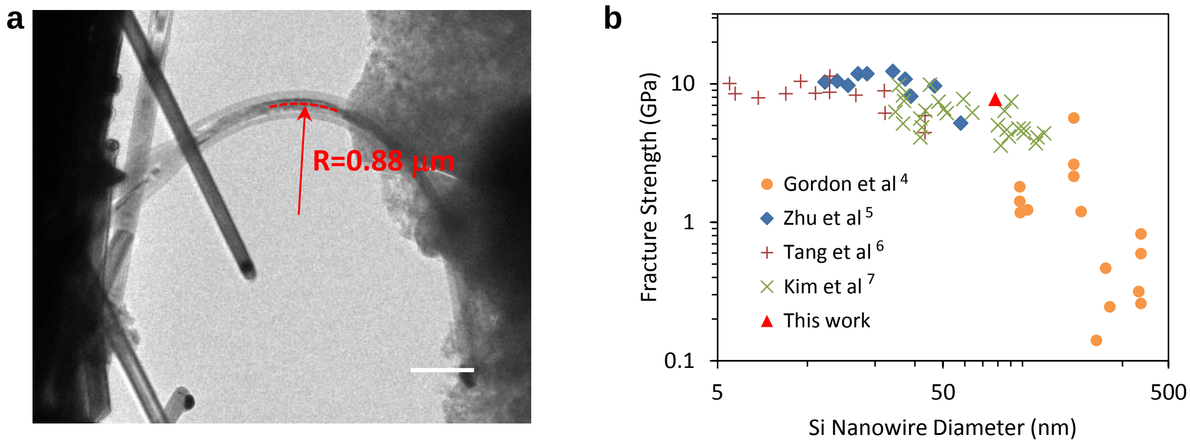

Supplementary Figure 1: (a) TEM image of a partially lithiated Si nanowire under bending. The image was taken right before brittle failure in the *c*-Si core (scale bar, 500 nm). (b) Comparison of the failure strength of the *c*-Si core with the literature data on the failure strength of Si nanowires with various diameters.

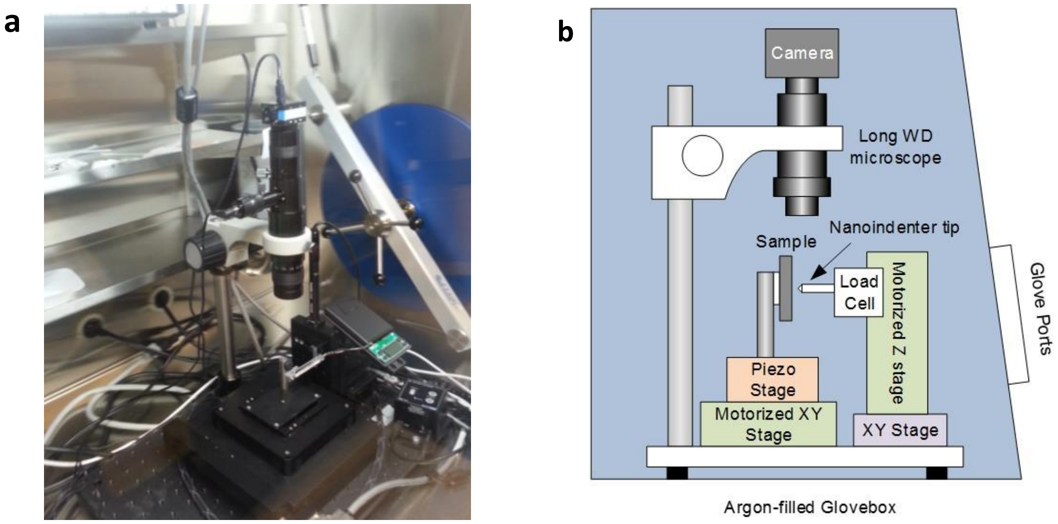

Supplementary Figure 2: (a) Photograph of an in-house developed nanoindenter for fracture toughness measurement. The nanoindenter is situated inside an argon-filled glove box to avoid exposure of the test material to ambient humidity and oxygen. (b) Schematic diagram of the nanoindenter.

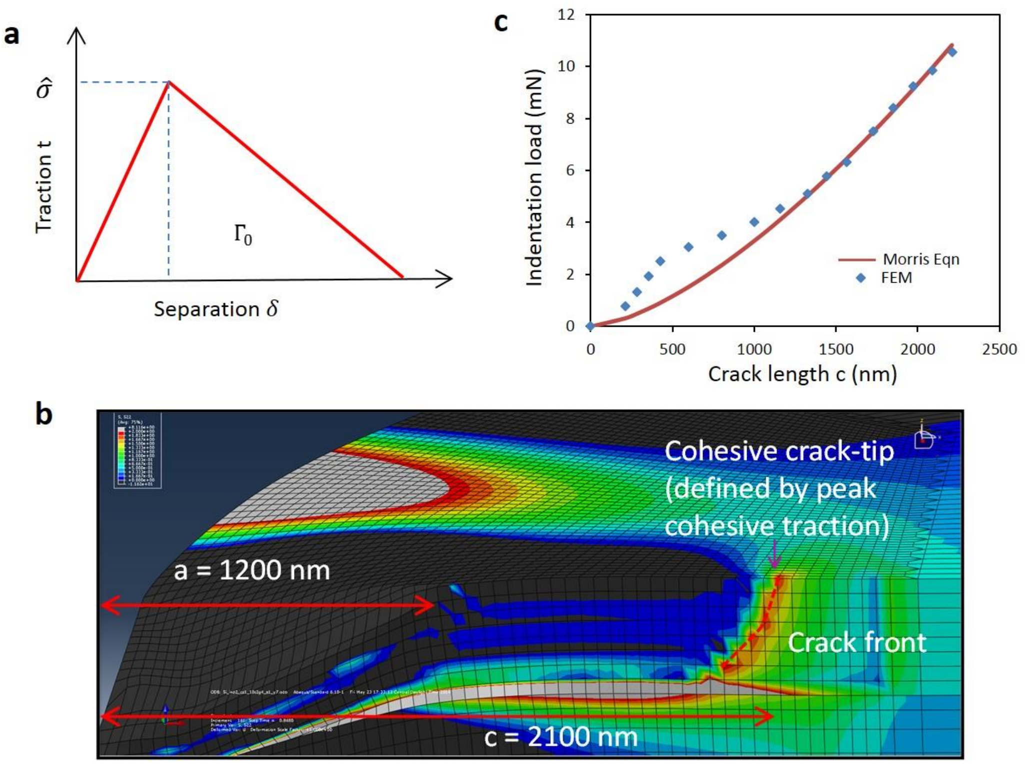

Supplementary Figure 3: (a) Illustration of traction-separation law. (b) Contour map of σ_{22} stresses during nanoindentation. (c) Comparison between the Morris model and finite element predictions about the indentation load as a function of surface crack length.

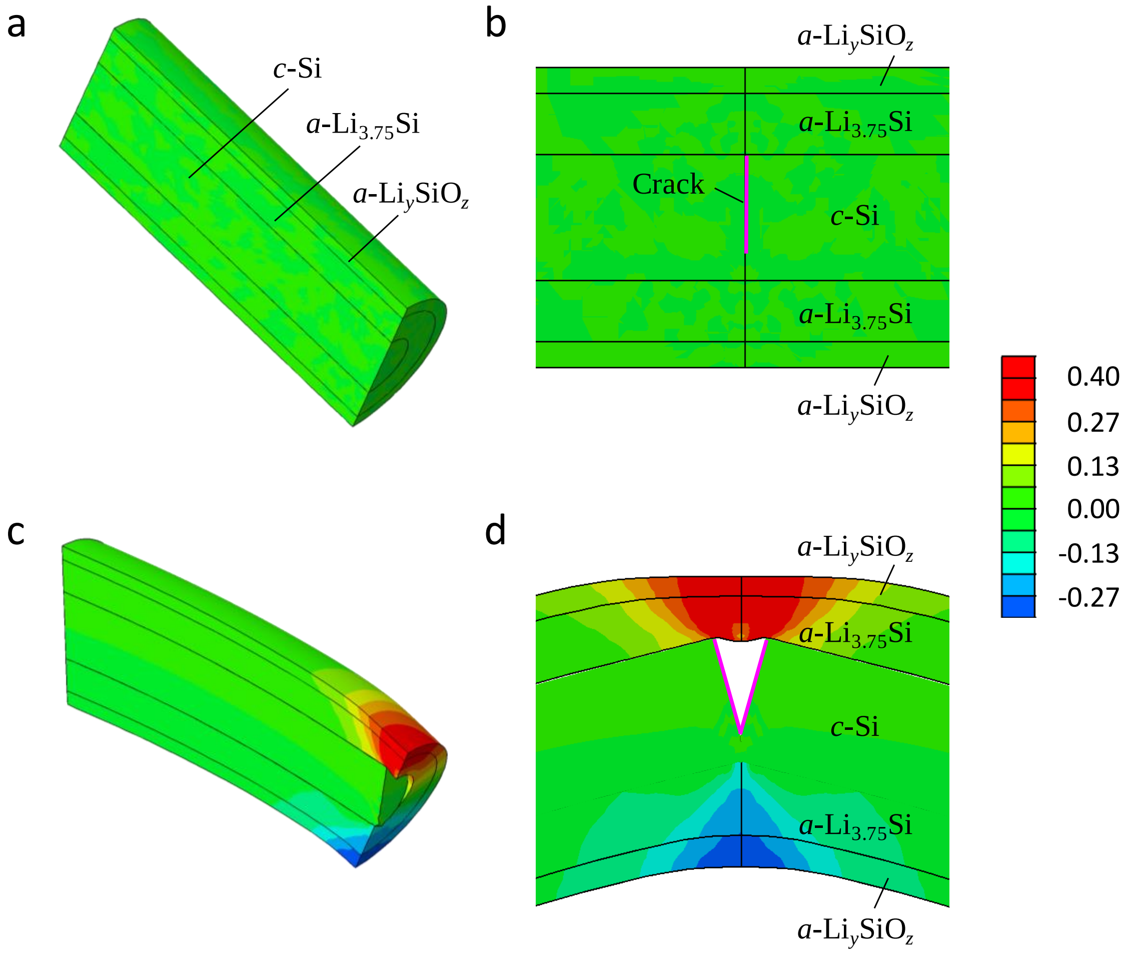

Supplementary Figure 4. Finite element simulation of bending of a lithiated Si nanowire. (a) 3D undeformed configuration consisting of a *c*-Si core, an *a*-$Li_{3.75}Si$ shell and an *a*-Li_ySiO_z coating. (b) 2D view of the longitudinal cross section in (a). A pre-crack is embedded in the *c*-Si core. (c) 3D deformed configuration with the color contour showing the distribution of axial strain. (d) 2D view of the longitudinal cross section in (c). This plot is identical to Fig. 1(e) in the paper.

Supplementary Methods

Failure strength of *c*-Si core

Our *in situ* TEM study shows that the lithiated shell of a bent *a*-Li_xSi/*c*-Si nanowire is highly ductile and deformable, while the *c*-Si core breaks in a brittle manner. There has recently been a wealth of studies concerning the mechanical testing of pure *c*-Si nanowires. It has been shown that the failure strength of *c*-Si nanowires is substantially higher than that of bulk Si. The work by Han et al.[1], Zheng et al.[2], and Wang et al.[3] showed that $\langle 110 \rangle$-oriented Si nanowires could undergo large plastic deformation under tensile or bending loads. However, for $\langle 111 \rangle$-oriented Si nanowires like the ones investigated in our work, the mode of deformation and failure remains brittle for a wide range of nanowire diameters from 500 nm down to 20 nm[4-7], which is consistent with the high brittleness of the *c*-Si core as observed in our work. For further verification, we calculated the failure strength of the *c*-Si core and compared it with the literature values. Since the *c*-Si core first breaks near the *a*-Li_xSi/*c*-Si interface where the largest tensile stress within the *c*-Si core occurs, we can determine the failure strength according to the beam bending equation as

$$\sigma_{\mathrm{Fail}} = \frac{ED}{2R_{\mathrm{Crit}}}, \qquad (1)$$

where *D* is the diameter of the *c*-Si core, *E* is the Young's modulus, and R_{Crit} is the critical radius of curvature of the neutral axis right before the brittle failure occurs. The critical radius of curvature R_{Crit} = 0.88 μm was obtained by extracting the profile of the neutral axis with the ImageJ software (National Institute of Health, Bethesda, MD) and fitting it to a parabolic curve (see Supplementary Fig. 1(a)). Taking *D* = 85 nm and *E* = 160 GPa[8], the failure strength of the *c*-Si core was determined to be about 7.7 GPa. Supplementary Fig. 1b summarizes the literature values for the failure strength of $\langle 111 \rangle$-oriented Si nanowires, together with the strength of the *c*-Si core obtained in our work. The literature strength values show a strong size-dependence, with smaller nanowires exhibiting higher failure strength. The strength of the brittle *c*-Si core from our work is seen to conform well to the literature data.

Nanoindentation system for fracture toughness measurement

An in-house developed nanoindentation system situated inside an argon-filled glove box, as shown in Supplementary Fig. 2, was employed to measure the fracture toughness of lithiated Si electrodes. A load cell with a capacity of 98 mN and a resolution of 0.01 mN was used to monitor the indentation load. A motorized stage with a positioning resolution of 0.1 μm was used for sample positioning. A piezo nanopositioning stage with a resolution of 0.1 nm was used for tip-sample engagement and disengagement. Fracture indentation tests were performed using a cube-corner diamond tip and monitored by a long working distance (WD) microscope.

Morris model for fracture toughness evaluation

The fracture toughness of lithiated Si electrodes was evaluated using the Morris Model[9, 10]. In this model, the radial crack evolution induced by acute cube-corner indenter geometry is governed by a pre-existing film-stress field and the elastic field of the indenter. The elastic field is composed of an elastic contact component and a “wedging” component, which is so named because the indenter pries open the radial cracks like a wedge. The fracture toughness of the test material is comprised of three components arising from these stress fields:

$$K_{IC} = K_{IC}^{F} + K_{IC}^{E} + K_{IC}^{W}. \tag{2}$$

The three components are calculated by the following formulas:

$$K_{IC}^{F} = \Psi\sigma\left(h_f\right)^{1/2}, \tag{3}$$

$$K_{IC}^{E} = \chi^{E}\frac{P}{c^{3/2}}\left[\frac{c}{a}\right], \tag{4}$$

$$K_{IC}^{W} = \chi^{W}\frac{P}{c^{3/2}}\left[\frac{c}{a} + \frac{a}{c}\ln\frac{2c}{a}\right], \tag{5}$$

where K_{IC}^{F} is the stress intensity factor from the biaxial film stress, and K_{IC}^{E} and K_{IC}^{W} are the stress intensity factors from the elastic-contact and wedging stress fields. P is the peak indentation load, and

χ^W and χ^E are two semi-empirical constants relating the wedging and elastic-contact components to the probe acuity and the Poisson's ratio of the material. Ψ is a factor related to the elastic mismatch ratio between the substrate and the film. Parameter a refers to the center-to-corner distance of the indent, and c refers to the average radial crack length measured from the center of the indent to the ends of the radial cracks.

Finite element simulation for parameter calibration in the Morris Model

To obtain the semi-empirical constants χ^W and χ^E required for fracture toughness evaluation in the Morris Model, finite element simulation of the nanoindentation process was carried out using the commercial finite element package ABAQUS 6.10. Both the amorphous Si film and the Ti substrate are assumed to be elastically isotropic, with elastic modulus of $E_{\text{Si}} = 92$ GPa and $E_{\text{Ti}} = 100$ GPa, and Poisson's ratio of $\nu_{\text{Si}} = 0.26$ and $\nu_{\text{Ti}} = 0.31$. The plastic responses of the Si film and the Ti substrate are characterized by a J_2 flow theory, and undergo perfectly plastic yielding at yield strengths of $\sigma_0^{\text{Si}} = 7$ GPa and $\sigma_0^{\text{Ti}} = 0.88$ GPa. Due to the three-fold rotational symmetry of the cube corner indenter, only one-six of the entire Si-Ti film-substrate geometry is modeled in the finite element method (FEM). Roller boundary conditions are imposed along the symmetric planes of the FEM model, i.e. along $x_2 = 0$ and $x_2 = \sqrt{3}x_1$. The Si film is modeled to have a uniform thickness of $h = 350$ nm, while the thickness of the Ti substrate is modeled to be $10h$, which effectively represents an infinitely thick substrate. The base of the Ti substrate is rigidly clamped. The far-field boundary of the film-substrate model away from the indenter-tip is taken to be traction-free. The cube-corner indenter is modeled using an analytically rigid surface, and the interaction between the rigid indenter and the Si-Ti thin film configuration is governed by a contact algorithm. The FEM mesh comprises of ~120,000 8-noded 3-dimensional brick elements, with uniform mesh density in the vicinity of the indenter tip, and a graded mesh away from this zone of interest. Along the crack-plane of the 350 nm thick Si film (x_2 = 0), the fracture process zone is modeled using 8-noded 2-dimensional cohesive elements, which are governed by the uncoupled traction-separation

law shown in Supplementary Fig. 3a. We have performed numerical tests by iteratively varying the peak cohesive traction $\hat{\sigma}$, critical separation distance δ_c, as well as the intrinsic fracture toughness Γ_0 (defined by the area enclosed by the traction-separation relationship), and monitoring the corresponding contact pressure *p*, indentation contact radius *a*, and surface crack length *c*. We have found the parameters of $\hat{\sigma} = 2$ GPa, $\delta_c = 0.75$ nm, and $\Gamma_0 = 1.5$ J m^{-2} to give an indentation contact radius of $a = 1200$ nm and a surface crack length of $c = 2100$ nm at the indentation load of $p = 9.8$ mN (Supplementary Fig. 3b), which fits well with our experimental values for amorphous Si. Note that these parameters are for a half-cohesive zone since only one-half of the crack is modeled, implying that the amorphous Si film has an intrinsic fracture toughness of 3 J m^{-2} in the absence of residual stress which gives a plane strain K_{IC} value of $0.544\ \mathrm{MPa}\sqrt{\mathrm{m}}$. Substituting these values in Eqns. (2)-(5) and assuming $-\chi_{CH}^{W}/\chi_{CH}^{E} = 1.95$, we obtain $\chi_{CH}^{E} = -0.080$ and $\chi_{CH}^{W} = 0.156$. Comparison between the indentation load versus surface crack length predictions of the Morris model and our FEM results using these calibrated stress-intensity-factor amplitude coefficients in Supplementary Fig. 3c demonstrates that the Morris model is quantitatively accurate at large indentation depths exceeding the film thickness.

Finite element simulations of bending test of a lithiated core-shell Si nanowire

Continuum finite element simulations are performed using the non-linear finite element package ABAQUS, in order to understand the *in situ* TEM observation of large tensile deformation in the *a*-$Li_{3.75}Si$ shell occurring during the bending of a lithiated Si nanowire. As shown in Supplementary Figs. 4(a, c), the 3D finite element model consists of a Si core, an *a*-$Li_{3.75}Si$ shell and an *a*-Li_ySiO_z layer of coating. A pre-crack is introduced in the cross section of the Si core, based on the assumption that the fast brittle fracture occurred before the tensile deformation in the *a*-$Li_{3.75}Si$ shell. We use the constitutive model that has been described in detail in our previous publication[11]. Specifically, the Si core is modeled as a linear elastic material, while both the *a*-$Li_{3.75}Si$ shell and the *a*-Li_ySiO_z coating as the elastic-plastic material; low strain hardening is introduced in both the shell and coating to facilitate numerical stability. It should be noted that the axial deformation in the fractured Si core is negligibly small, while the axial

elongation in the *a*-$Li_{3.75}Si$ shell is extremely large at ~ 40%. Hence, there is a large deformation mismatch at the interface between the fractured Si core and the *a*-$Li_{3.75}Si$ shell. At the moment of this study, it is unclear how such large deformation mismatch was accommodated, e.g., through sliding at the interface, or plastic flow in the *a*-$Li_{3.75}Si$ shell near the interface, or possibly both. Nonetheless, we treat the effective interface sliding by using the Cohesive Surface Contact option in ABAQUS[12], which maintains the normal contact between the contact surfaces, but allows the relative sliding across the interface through a bi-linear traction-displacement constitute law (i.e., initially linear-elastic and subsequently linear-softening traction-displacement laws). Considering the symmetry conditions of nanowire geometry and bending deformation, we model one half of the nanowire as shown in Supplementary Fig. 4(a). The bending load is applied through a prescribed rotation at the left end of the nanowire, while the uncracked mid-section of the nanowire is fixed in the axial direction. To capture the key deformation features observed in the TEM image, particularly, the curved shape of the exposed inner surface of the *a*-$Li_{3.75}Si$ shell, we apply the bending load through an over-loading and unloading procedure, so as to mimic the over-bending and spring-back that presumably occurred during the buckling deformation of the lithiated nanowire. The finite element simulations are performed using the explicit numerical procedure in ABAQUS Explicit[12].

Supplementary References

1. Han, X., Zheng, K., Zhang, Y., Zhang, X., Zhang, Z., Wang, Z. L. Low-temperature in situ large-strain plasticity of silicon nanowires. *Advanced Materials* **19**, 2112 (2007).
2. Zheng, K. *et al.* Atomic mechanisms governing the elastic limit and the incipient plasticity of bending Si nanowires. *Nano Letters* **9**, 2471-2476 (2009).
3. Wang, L., Zheng, K., Zhang, Z., Han, X. Direct atomic-scale imaging about the mechanisms of ultralarge bent straining in Si nanowires. *Nano Letters* **11**, 2382-2385 (2011).
4. Gordon, M. J., Baron, T., Dhalluin, F., Gentile, P., Ferret, P. Size effects in mechanical deformation and fracture of cantilevered silicon nanowires. *Nano Letters* **9**, 525-529 (2009).
5. Zhu, Y., Xu, F., Qin, Q., Fung, W. Y., Lu, W. Mechanical properties of vapor− liquid− solid synthesized silicon nanowires. *Nano Letters* **9**, 3934-3939 (2009).
6. Tang, D.-M. *et al.* Mechanical properties of Si nanowires as revealed by in situ transmission electron microscopy and molecular dynamics simulations. *Nano Letters* **12**, 1898-1904 (2012).
7. Kim, Y. J., Son, K., Choi, I. C., Choi, I. S., Park, W. I., Jang, J. i. Exploring nanomechanical behavior of silicon nanowires: AFM bending versus nanoindentation. *Advanced Functional Materials* **21**, 279-286 (2011).
8. Stan, G., Krylyuk, S., Davydov, A., Cook, R. Compressive stress effect on the radial elastic modulus of oxidized Si nanowires. *Nano Letters* **10**, 2031-2037 (2010).
9. Morris, D. J., Cook, R. F. Indentation fracture of low-dielectric constant films: Part II. Indentation fracture mechanics model. *J Mater Res* **23**, 2443-2457 (2008).

10. Morris, D. J., Cook, R. F. Indentation fracture of low-dielectric constant films: Part I. Experiments and observations. *J Mater Res* **23**, 2429-2442 (2008).

11. Huang, S., Fan, F., Li, J., Zhang, S. L., Zhu, T. Stress generation during lithiation of high-capacity electrode particles in lithium ion batteries. *Acta Materialia* **61**, 4354-4364 (2013).

12. ABAQUS/Standard 6.10, User's Manual. *SIMULIA*, Providence, RI (2010).
